# Impact of estrogen receptor expression on prognosis of ovarian cancer according to antibody clone used for immunohistochemistry: a meta-analysis

**DOI:** 10.1186/s13048-022-01001-4

**Published:** 2022-05-24

**Authors:** Chun Wai Ng, Kwong-Kwok Wong

**Affiliations:** 1 Department of Gynecologic Oncology & Reproductive Medicine, Room T4-3900, Clinical Research Building, The University of Texas MD Anderson Cancer Center, 1515 Holcombe Boulevard, Houston, TX 77030 USA; 2 The University of Texas MD Anderson Cancer Center UTHealth Graduate School of Biomedical Sciences, Houston, TX USA

**Keywords:** Antibody, Biomarker, Estrogen receptor, Meta-analysis, Ovarian cancer, Prognosis, Subtype

## Abstract

**Background:**

The prognostic value of the expression of estrogen receptor (ER) subtypes ER⍺ and ERβ in ovarian cancer has previously been evaluated by meta-analyses. However, the results are contradictory and controversial.

**Methods:**

We conducted an updated meta-analysis with stringent inclusion criteria to ensure homogeneous studies to determine the effect of ER subtypes on ovarian cancer prognosis. Articles were retrieved by systematic search of PubMed and Web of Science for articles dated up to June 2021. Only studies with known hazard ratio (HR) and antibody clone for immunochemistry (IHC) were included. Pooled HRs with the corresponding 95% confidence intervals (CIs) were calculated for the effect of ER⍺ and ERβ expression on ovarian cancer patient progression-free survival (PFS) and overall survival (OS).

**Results:**

A total of 17 studies were included, of which 11 and 13 studies examined the relationships between ER⍺ expression and PFS and OS, respectively, and 5 and 7 studies examined the relationships between ERβ expression and PFS and OS, respectively. Neither ER⍺ expression (random-effects model; HR = 0.99, 95% CI = 0.83–1.18) nor ERβ expression (fixed-effects model; HR = 0.94, 95% CI = 0.69–1.27) was associated with PFS. Random-effects models showed that ER⍺ expression (HR = 0.81, 95% CI = 0.64–1.02) and ERβ expression (HR = 0.75, 95% CI = 0.50–1.13) were only marginally and not significantly associated with better OS. Subgroup analysis revealed that ER⍺ expression determined using antibody clone 1D5 (HR = 0.75, 95% CI = 0.64–0.88) and ERβ expression determined using ERβ1-specific-antibody clone PPG5/10 or EMR02 (HR = 0.65, 95% CI = 0.50–0.86) were associated with significantly better OS, but ER expression determined using other antibodies was not.

**Conclusions:**

In conclusion, a higher ER⍺ expression and ERβ expression are significantly associated with a better survival of ovarian cancer patients, but the results from previous prognostic studies are significantly dependent on the choice of specific ER antibody clones used in immunohistochemistry analysis.

## Background

Ovarian cancer is one of the top five causes of death from gynecological cancer in developed countries [1]. Because of the lack of effective early diagnostic methods and aggressive behavior, ovarian cancer is usually detected at late stages and has a low survival rate (10–30% 5-year survival rate) [2]. In 2020, about 21,750 cases were diagnosed and 13,940 individuals died from ovarian cancer in the US [3]. Approximately 90% of ovarian cancer cases are classified as epithelial ovarian cancer. Given this situation, discovering biomarkers for prognosis, response to therapeutic intervention, and development of novel treatment strategies is desperately needed.

Estrogen receptor (ER) and its ligand estrogen have long been recognized to play important roles in ovarian cancer [4]. ER signaling has been shown to be oncogenic by promoting cancer cell survival and proliferation [5]. Two subtypes of ER have been identified, ER⍺ and ERβ [6–8]. ER⍺, also named ESR1, was first identified in the 1950s by Jensen and Jordan [9]. Approximately 50% of ovarian tumor tissues express ER⍺ [10]. ERβ, also named ESR2, was identified by Kuiper et al. in 1996 [8]. In vitro experiments showed that ER expression is responsible for ovarian cancer cell growth. Anti-estrogens, which can inhibit the interactions between ER and estrogen, were shown to inhibit ovarian cancer cell growth [11]. Examination of clinical specimens and in vitro experiments showed that high expression of ER⍺ was associated with a better response to anti-estrogen treatment [12]. ERβ is also a key factor in ovarian cancer pathogenesis and associated with responsiveness to hormonal treatment in ovarian cancer [13].

Given the important role of ER signaling in ovarian cancer, studies have interrogated the relationship between ER expression and ovarian cancer prognosis [14]. ER expression was expected to be related to better prognosis, as is the case in breast cancer [15, 16]. However, contradictory results were reported. While Bizzi, Codegoni [17] and Yang, Xi [18] found that ER expression was linked with better prognosis of patients with epithelial ovarian cancer, Liew, Hsu [19] found that ER expression did not affect the prognosis of patients with epithelial ovarian cancer. Khandakar, Mathur [20] reported an inverse association between ER expression and epithelial ovarian cancer patients’ survival.

The proportion of ER-positive ovarian cancer cases that respond to anti-estrogens such as tamoxifen is low (< 10%) compared to the proportion of breast cancer cases that respond (~ 80%) [14, 21]. This leads to questions about the function of ER signaling in ovarian cancer. Mechanisms that may explain the lower responsiveness of ER-positive ovarian cancer than breast cancer to anti-estrogens include differences in 1) the expression of the subtypes of ER, 2) the expression of ER coactivators, and 3) expression patterns of ER isoforms. Recent studies have found that whereas ER⍺ acts as a tumor promoter in ovarian cancer, ERβ acts as a tumor suppressor in ovarian cancer [22–24].

Given the unexplained heterogeneity of the results from previously published meta-analyses [25, 26], the specific antibody used to measure ER expression should be taken into consideration in examinations of the impact of ER expression on ovarian cancer patients’ survival. Although the predictive and prognostic values of different ER antibodies have been extensively tested in breast cancer, they have not yet been determined in ovarian cancer [27–30]. Therefore, to gain insight into the prognostic value of ER expression in ovarian cancer and guide future research, we conducted an updated meta-analysis that included only studies with ER subtypes determined by immunohistochemistry (IHC) with known specific antibody.

## Results

### Literature search results

**Fig. 1:**
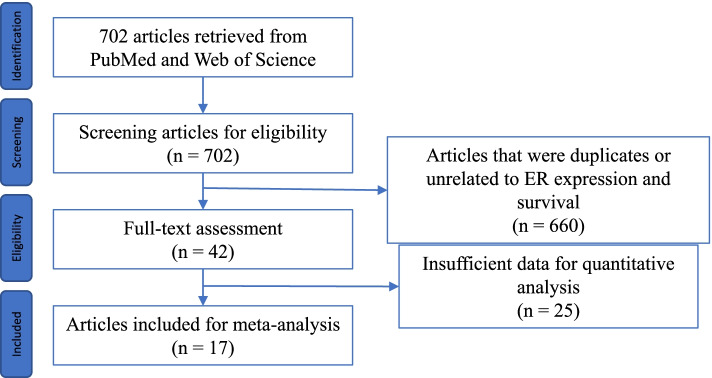
Selection of articles for the meta-analysis

From the initial search in PubMed and Web of Science, 702 articles were retrieved. After examination of the title and abstract, 660 articles that were duplicate or obviously irrelevant to the topics of survival and ER expression were excluded. From the remaining 42 articles, the full text was evaluated, and articles were included if ER expression was determined by IHC, ER⍺ and/or ERβ/ERβ1 was examined, the antibody clone was specified, and a HR was provided for OS and/or PFS. Finally, 17 articles were included for meta-analysis. The article selection process is described in Fig. 1.

### Characteristics of the included studies

The 17 articles included for this meta-analysis are summarized in Table 1. A total of 6172 patients were included. The majority of the studies included patients with a mixture of epithelial ovarian cancer subtypes. Approximate proportions of patients with the different subtypes were as follows: serous, 4082 (66%); endometrioid, 913 (15%); clear cell, 486 (8%); mucinous, 466 (8%); mixed epithelial, 60 (1%); undifferentiated, 45 (1%); and other (carcinosarcoma, adenocarcinoma, transitional, and unknown epithelial), 120 (2%). The antibody clones employed for detection of ER⍺ were 1D5, 6F11, and SP1, and the antibody clones employed for detection of ERβ were PPG5/10, EMR02 and 14C8.

**Table 1:** Studies included in the meta-analysis

Study	Region	No. of Patients	Mean/Median Age, y	ER⍺ Antibody Clone (Dilution)	ERβ Antibody Clone (Dilution)	Outcome Analyzed (ER Subtype)
Aust, Bachmayr-Heyda [31]	Austria	101	56 (Median)	1D5 (1:50)	ND	PFS (ER⍺) and OS (ER⍺)
Aust, Horak [32]	Austria	63	58.3 (Median)	1D5 (1:50)	PPG5/10 (1:20)	PFS (ERβ) and OS (ERβ)
Battista, Mantai [33]	Germany	107	61.7 (Mean)	1D5 (NR)	ND	PFS (ER⍺)
Burges, Brüning [34]	Germany	100	60.35 (Mean)	1D5 (1:150)	PPG5/10 (1:50)	PFS (ER⍺) and OS (ER⍺ and ERβ)
Chan, Wei [35]	Hong Kong	173	50 (Mean)	1D5 (1:100)	EMR02 (1:30)	OS (ERβ)
De Sousa Damião, Fujiyama Oshima [36]	Brazil	85	55.8 (Mean)	6F11 (1:50)	ND	OS (ER⍺)
De Stefano, Zannoni [37]	Italy	58	54 (Median)	6F11 (1:100)	14C8 (1:30)	PFS (ER⍺ and ERβ) and OS (ER⍺ and ERβ)
de Toledo, Sarian [38]	Brazil	152	55.2 (Mean)	1D5 (1:1000)	14C8 (1:600)	PFS (ER⍺ and ERβ) and OS (ER⍺ and ERβ)
Feng, Wen [39]	China	875	56 (Median)	SP1 (NR)	ND	PFS (ER⍺) and OS (ER⍺)
Jönsson, Arildsen [40]	Sweden	35	58 (Median)	1D5 (1:100)	PPG5/10 (1:10)	PFS (ER⍺ and ERβ) and OS (ER⍺ and ERβ)
Kjaer, Christensen [41]	Denmark	720	NR	1D5 (1:200)	ND	OS (ER⍺)
Lee, Rosen [42]	USA	258	58.3 (Mean)	6F11 (1:50)	ND	OS (ER⍺)
Liew, Hsu [19]	China	108	53 (Median)	6F11 (NR)	ND	PFS (ER⍺) and OS (ER⍺)
Llaurado Fernandez, Dawson [43]	British	100	48.5 (Mean)	SP1 (NR)	ND	PFS (ER⍺) and OS (ER⍺)
Rambau, Kelemen [44]	Calgary	182	54 (Mean)	SP1 (1:50)	ND	OS (ER⍺)
Sieh, Köbel [45]	USA	2933	60.9 (Mean)	SP1 (1:25)	ND	PFS (ER⍺)
van Kruchten, van der Marel [46]	Netherlands	121	61 (Median)	SP1 (NR)	14C8 (NR)	PFS (ER⍺ and ERβ) and OS (ER⍺ and ERβ)

*NR* not reported, *ND* IHC for ERβ not done

### Associations between ER⍺ and PFS and OS

**Fig. 2:**
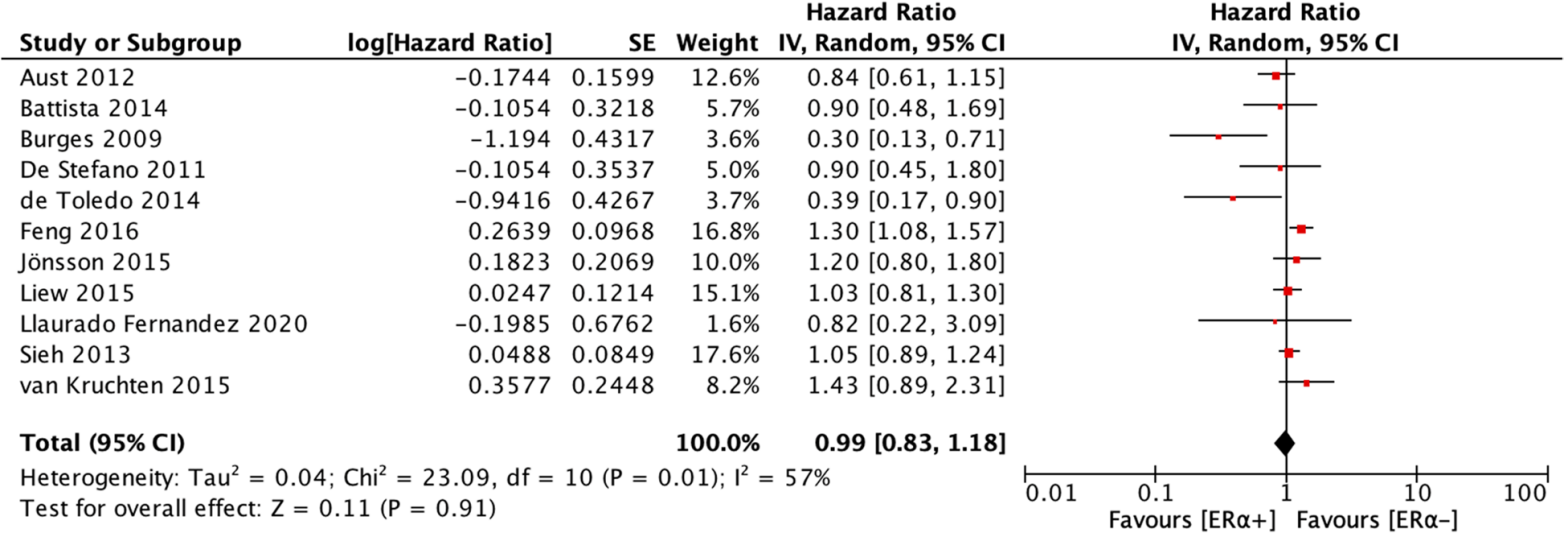
Forest plot (HR and 95% CI) of meta-analysis of impact of ER⍺ expression on PFS

**Fig. 3:**
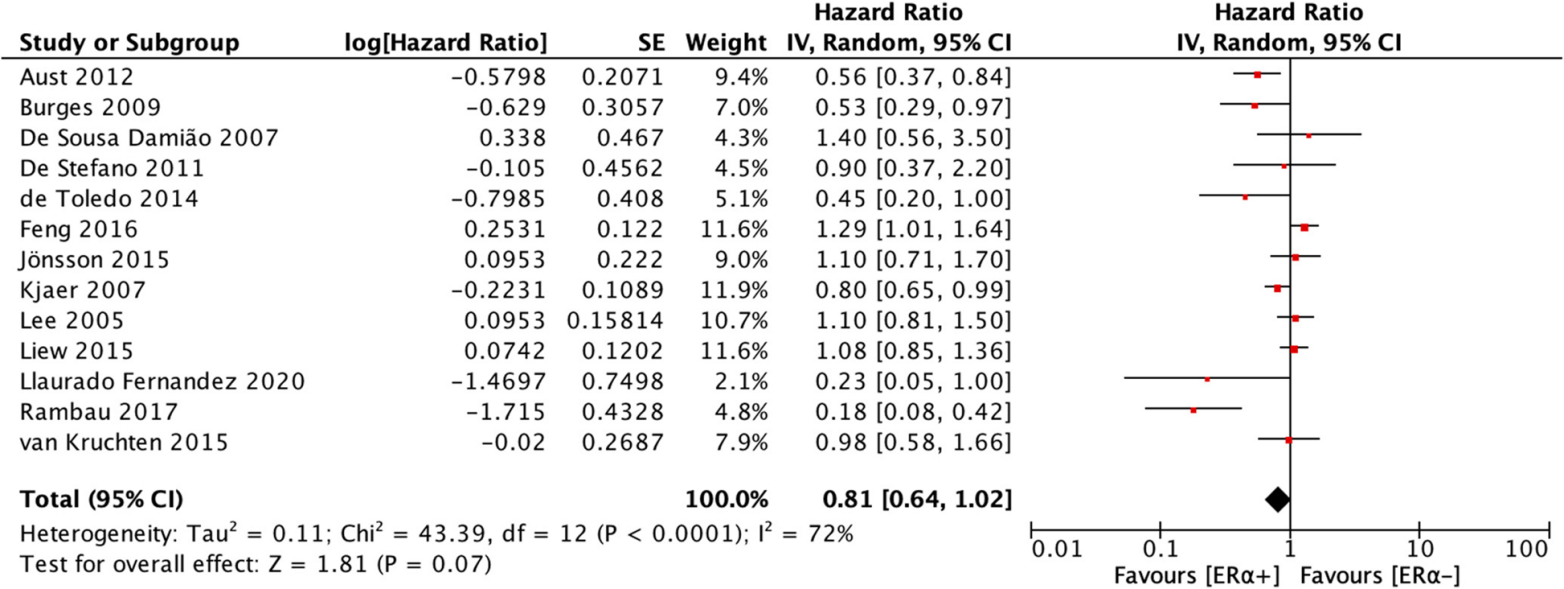
Forest plot (HR and 95% CI) of meta-analysis of impact of ER⍺ expression on OS

The analyses of the relationships between ER⍺ expression and PFS and OS of patients with ovarian cancer included 11 studies and 13 studies, respectively. Heterogeneity was moderate for the studies included in the PFS analysis (I^2^ = 57%, *p* = 0.01) and the studies included in the OS analysis (I^2^ = 72%, *p* < 0.0001). Therefore, a random-effects model was employed to calculate the pooled HRs for both PFS and OS. ER⍺ expression was not associated with PFS (HR = 0.99, 95% CI = 0.83–1.18) (Fig. 2) but was significantly associated with better OS (HR = 0.81, 95% CI = 0.64–1.02) (Fig. 3).

**Fig. 4:**
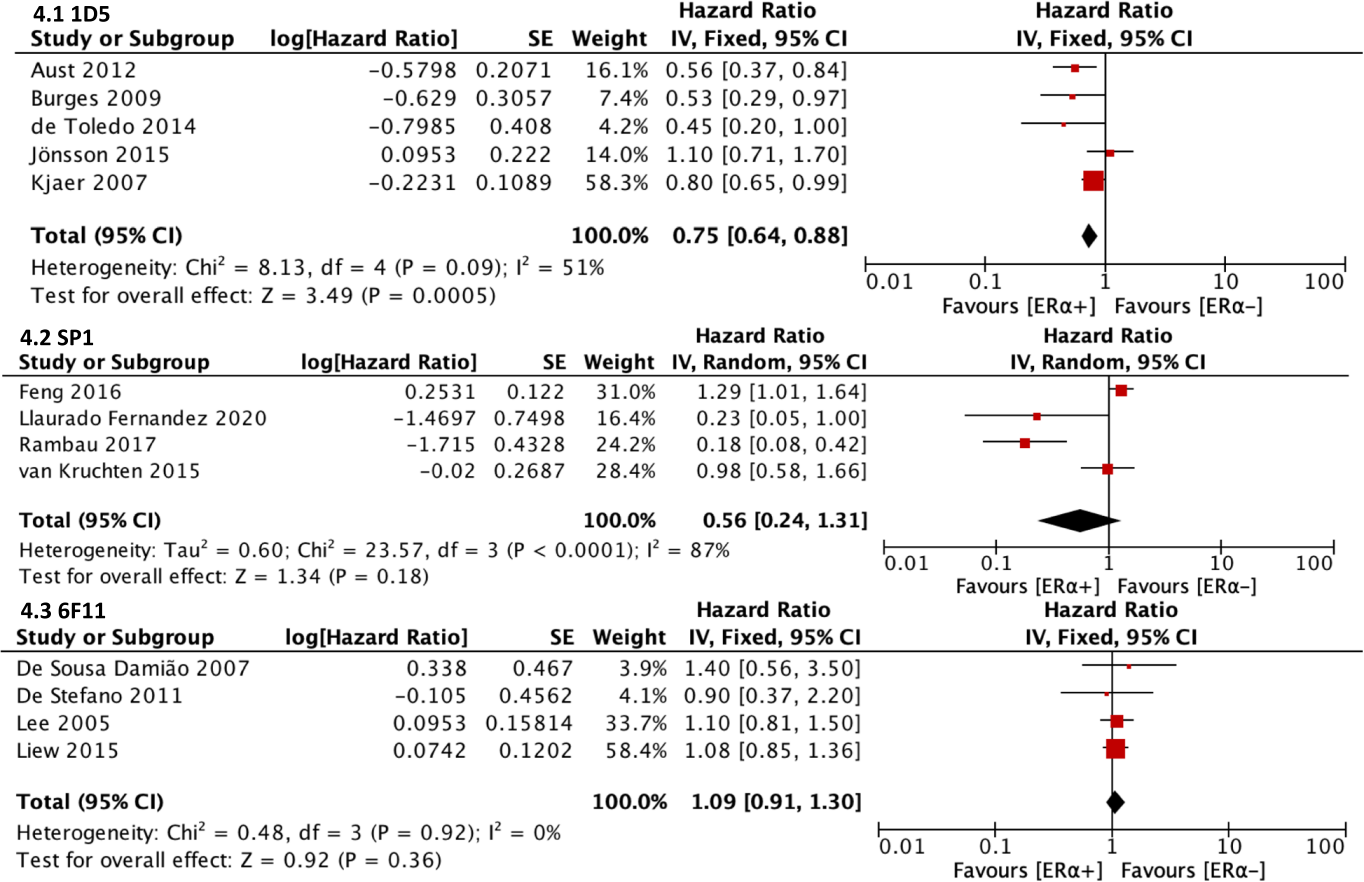
Forest plot (HR and 95% CI) of the subgroup meta-analysis of ER⍺ expression and OS

Subgroup analysis was then done for the effect of ER⍺ expression on OS by ER⍺ antibody clone. Studies using clone 1D5 showed that ER⍺ expression was significantly associated with better OS (HR = 0.75, CI = 0.64–0.88), while studies using clones SP1 (HR = 0.56, CI = 0.24–1.31) and 6F11 (HR = 1.09, CI = 0.91–1.30) did not (Fig. 4).

### Associations between ERβ and PFS and OS

**Fig. 5:**
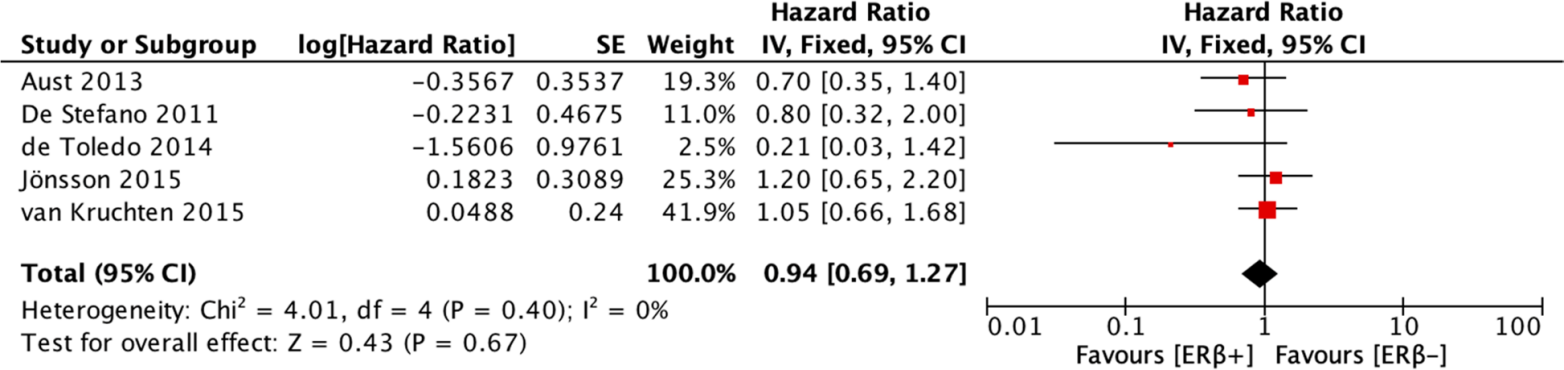
Forest plot (HR and 95% CI) of meta-analysis of impact of ERβ expression on PFS

**Fig. 6:**
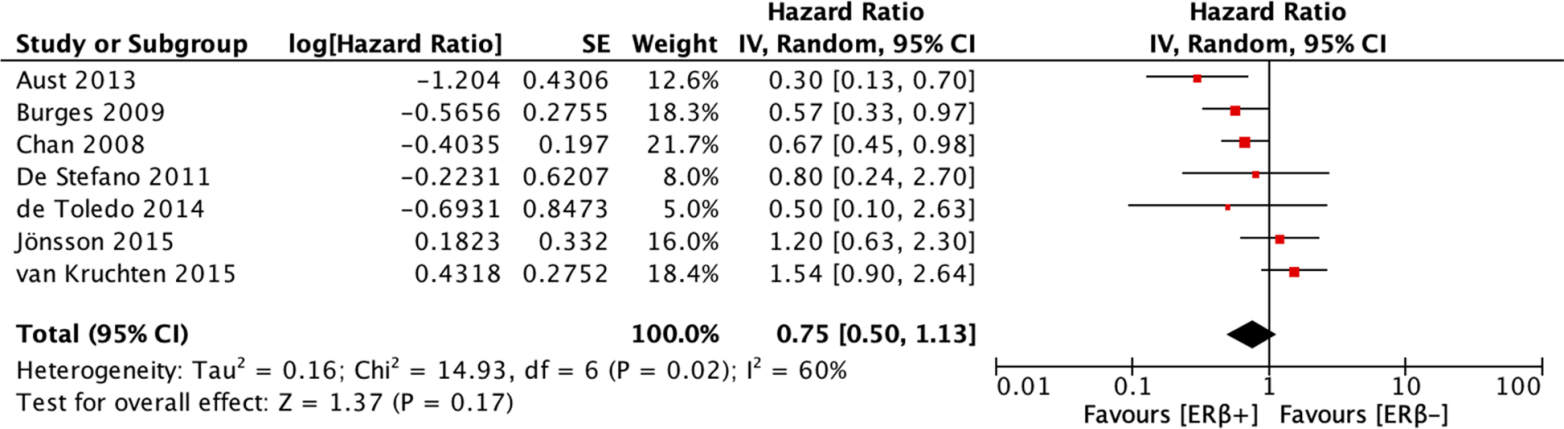
Forest plot (HR and 95% CI) of meta-analysis of impact of ERβ expression on OS

The analyses of the relationships between ERβ expression and PFS and OS of patients with ovarian cancer included 5 studies and 6 studies, respectively. Heterogeneity was low for the studies included in the PFS analysis (I^2^ = 0%, *p* = 0.40) and moderate for the studies included in the OS analysis (I^2^ = 60%, *p* = 0.02). Therefore, a fixed-effects model was employed to calculate the pooled HR for PFS, and a random-effects model was employed to calculate the pooled HR for OS. ERβ expression was not associated with PFS (HR = 0.94, CI = 0.69–1.27) (Fig. 5) or OS (HR = 0.75, CI = 0.50–1.13) (Fig. 6).

**Fig. 7:**
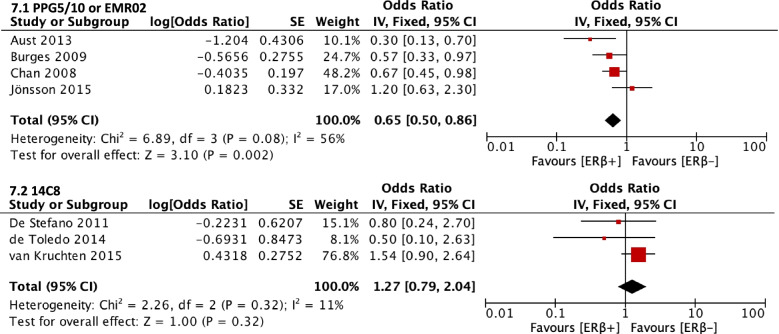
Forest plot (HR and 95% CI) of the subgroup meta-analysis of ERβ expression and OS

Subgroup analysis was then done for the effect of ERβ expression on OS by ERβ antibody clone. Studies using clone PPG5/10 or EMR02 (both known for targeting ERβ1) (HR = 0.65, CI = 0.50–0.86) showed that ER⍺ expression was significantly associated with better OS, while studies using clone 14C8 (HR = 1.27, CI = 0.79–2.04) did not (Fig. 7).

## Discussion

This meta-analysis showed that ER⍺ expression and ERβ expression determined using certain antibody clones were each associated with OS in patients with ovarian cancer.

Systematic reviews and meta-analyses have previously been done to determine the effects of ER expression on clinical outcomes of patients with ovarian cancer [25, 26]; however, none of these studies addressed the use of different detection methods and different antibodies in different studies. To address the weaknesses of the previous studies, we limited our analysis to studies for which HR was reported, ER expression was determined by IHC, and the specific antibody clones used were specified. Although this approach could reduce the power of the analysis, it could also improve the accuracy, analyzability, and interpretability of the results by only including articles with information that has important clinical implications.

HR is the most commonly used parameter for comparing the odds of survival over a period of time between two groups, we opted to use HR for our meta-analysis. As stated in reports of the previous meta-analyses, the indirect extraction of HR might reduce the accuracy of the meta-analysis [25, 26]. Thus, we opted to include only studies with a reported HR for accuracy.

IHC is the standard method for assessing the expression of ER in the clinical setting [47] ER expression can also be determined using other methods, such as RT-PCR and dextran-coated charcoal method [48, 49]. However, the different methods differ in terms of sensitivity and specificity, and thus including studies with different ER detection methods could confound and add variability to the analysis. The results based on an analysis limited to studies using IHC can also add relevancy and applicability to clinical prognosis prediction directly, as IHC is used commonly in clinical setting.

IHC is the most common method for determining the expression of ER, and antibody selection is a critical determinant of the performance of IHC. Different clones of ER antibodies have been evaluated in detecting ER expression of breast cancer [50, 51]. However, similar evaluations have not been done in ovarian cancer. In our meta-analysis, we included only studies with known antibody clones for consistency. Different antibody clones have different sensitivity and specificity, which means that including studies with an unknown antibody clone would have left us unable to draw conclusions about how specific antibody clones might have influenced the results. In addition, further subgroup analysis of different clones of antibody could be done for studies with known antibodies.

The marginality of the association between the expression of ER⍺ and ERβ and better OS that we observed in this study might be due to the unexplained heterogeneity of the methods of the studies. The three ER⍺ antibodies used in the studies included in our meta-analysis, 1D5, SP1, and 6F11, were previously tested and shown to have good and similar prognostic value in breast cancer [52]. However, in our analysis, only clone 1D5 was associated with better OS in ovarian cancer. Furthermore, of the three ERβ antibodies used in the studies included in our meta-analysis, 14C8 and PPG5/10 (or EMR02), only PPG5/10 (or EMR02) was associated with better OS. These antibody-clone-based differences might be due to the preferential binding of ER isoforms by different clones. The ER⍺ antibodies 1D5 and 6F11 were induced by a full-length ER⍺ protein (66 kDa ER⍺), while SP1 was induced by the C-terminus of ER⍺ that exists in the 46 kDa ER⍺ variant. Both clones 1D5 and 6F11 bind to the A/B domain of ER⍺ (only exists in 66 kDa ER⍺), the completely different results from the two clones might be due to detection sensitivity as (i.e., low sensitivity could only stain samples with high expression) [53–55]. The ERβ antibody PPG5/10 and EMR02 were induced by synthetic peptide derived from the C-terminus of the human ERβ, which only exists in ERβ isoform ERβ1 [56]. The ERβ antibody 14C8 was induced by the first 153 amino acids of ERβ1, which exists in all ERβ isoforms. ERβ has five alternatively spliced isoforms, ERβ1–5, and it may be that only expression of ERβ1 correlates with better OS in ovarian cancer [57]. Alternatively, some clones might have non-specific binding. This also suggests that the detection of isoforms other than wild-type ER could also confound the results.

In those studies that used the antibodies with significant results, serous ovarian cancer was the major subtype among their samples, and the reported mean/median ages of those studies were between 50 and 60.35. Most of those studies are from Western countries, except one from South American and one from Hong Kong. Also, the study of Jönsson, Arildsen [40] had relatively small sample size (*n* = 35). In the selected studies, multivariant Cox proportional-hazard model has been performed for prognostic factors in individual paper. Other covariates such as FIGO stage and age were also predictive of survival in some individual papers.

To our knowledge, this is the first study to show that the choice of antibody for ER staining could lead to a completely different result. Inconsistent methods and the lack of granularity in assessing the intensity of ER have offered as potential explanations for the inconsistent results of different studies, such as biomarker studies of the hormonal therapy based on ER expression in ovarian cancer [58, 59]. Our study reported here shows that the choice of antibodies also contributes to different results.

With the analysis approach in this study, our results are not consistent with the results of the previously published meta-analyses of the impact of ER status on ovarian cancer prognosis [25, 26]. Although our meta-analysis included fewer studies than the previous meta-analyses did, the studies that we included were more homogeneous.

A limitation of our study is that we estimated pooled HRs from studies that included different proportions of patients with different subtypes of ovarian cancer. Since different subtypes of ovarian cancer also have different expression levels of ERs and estrogen signaling mechanisms, the pooled HRs of ER expression cannot be assumed to agree with the HR for any particular subtype of ovarian cancer. Further studies focusing on certain subtype of ovarian cancer should be done.

## Conclusions

In conclusion, a higher ER⍺ expression and ERβ expression are significantly associated with a better survival of ovarian cancer patients, but the results from previous prognostic studies are significantly dependent on the choice of specific ER antibody clones used in IHC analysis.

## Methods

### Literature search strategy and inclusion criteria

We searched the literature databases PubMed and Web of Science. The articles from 1982 to June 2021 were searched. The terms (“estrogen” or “hormon*” or “steroid”) and “receptor*” and “ovarian” were queried for the title, and the terms (“clinical” or “survival” or “outcome”) were queried for all fields. Studies were included only if the article provided information about the hazard ratios (HRs) for progression-free survival (PFS) and/or overall survival (OS) based on ER⍺ and/or ERβ expression determined by IHC. Studies with fewer than 10 samples were excluded. In the selected papers, most papers defined disease-free survival (DFS) as time interval between primary surgery and recurrence, and progression-free survival (PFS) as time interval between diagnosis and progression. In some papers, DFS and PFS were used interchangeably. In this study, PFS is defined as the time between diagnosis/surgical-procedure and relapse or recurrence or progression of ovarian cancer. OS is defined as the time between diagnosis/surgical-procedure and disease-related death.

### Data collection and statistical analysis

Additional data extracted from the included studies included analysis outcomes, antibody used, and mean/median age. The heterogeneity of the studies was analyzed by Cochran’s Q and I^2^ tests [60]. A fixed-effects model was used if the heterogeneity was low, and a random-effects model was used if the heterogeneity was high. The pooled HRs between ER-negative and ER-positive for different subgroups were calculated and presented using forest plots. Meta-analyses were performed by using RevMan [61].

## Data Availability

The data were retrieved from published articles and is available from author upon request.

## Abbreviations

CI: Confidence interval
ER: Estrogen receptor
HR: Hazard ratio
IHC: Immunohistochemistry
OS: Overall survival
PFS: Progression-free survival
